# The gene signature of tertiary lymphoid structures within ovarian cancer predicts the prognosis and immunotherapy benefit

**DOI:** 10.3389/fgene.2022.1090640

**Published:** 2023-01-10

**Authors:** Yue Hou, Sijing Qiao, Miao Li, Xue Han, Xuan Wei, Yingxin Pang, Hongluan Mao

**Affiliations:** ^1^ Department of Obstetrics and Gynecology, Qilu Hospital of Shandong University, Jinan, Shandong, China; ^2^ Division of Gynecology Oncology, Qilu Hospital of Shandong University, Jinan, Shandong, China; ^3^ Key Laboratory of Gynecology Oncology of Shandong Province, Qilu Hospital of Shandong University, Jinan, Shandong, China; ^4^ Shandong Engineering Laboratory for Urogynecology, Qilu Hospital of Shandong University, Jinan, Shandong, China

**Keywords:** tertiary lymphoid structures, gene signature, prognosis, immunotherapy, ovarian cancer

## Abstract

Ovarian cancer (OC) has the lowest survival rate among gynecologic malignancies. Ectopic lymphocyte aggregates, namely tertiary lymphoid structures (TLSs), have been reported as positive biomarkers for tumor prognosis. However, the related gene signature of tertiary lymphoid structure in ovarian cancer was less understood. Therefore, this study first exhibited the organizational patterns of tertiary lymphoid structure by H&E staining and immunohistochemistry (IHC), and confirmed the improved survival values of tertiary lymphoid structure and quantified tumor-infiltrating lymphocytes (CD20^+^ B cells and CD8^+^ T cells) in ovarian cancer patients. Secondly, we collected the genes involved in tertiary lymphoid structure from databases. By the univariate regression analysis, the tertiary lymphoid structure gene signature (CETP, CCR7, SELL, LAMP3, CCL19, CXCL9, CXCL10, CXCL11, and CXCL13) with prognostic value, characteristically of ovarian cancer, was constructed in the TCGA dataset and validated in the GSE140082 dataset. Thirdly, by performing CIBERSORT and Tumor Immune Dysfunction and Exclusion (TIDE) analysis, we found that the high expression of this gene signature was positively correlated with developed immune infiltration and reduced immune escape. The improved IPS score and application in the IMvigor210 dataset received PD-L1 proved the predictive value of immunotherapy for this gene signature. Furthermore, this signature showed a better correlation between tumor mutation burden and classical checkpoint genes. In conclusion, Tertiary lymphoid structure plays important role in tumor immunity and the gene signature can be evaluated as a biomarker for predicting prognosis and guiding immunotherapy in ovarian cancer.

## 1 Introduction

Ovarian cancer is just like a “secluded killer,” menacing the health of the female reproductive system. The classical treatment regimens for ovarian cancer focus on tumor-reducing surgery and platinum-based chemotherapy (Marchetti et al., 2021). However, accounting for tardy diagnosis, extensive metastasis, recurrence, and resistance to chemotherapy drugs, the 5-year survival rate is less than 50% (Torre et al., 2018; Yang et al., 2018; An and Yang 2021). The poly-ADP-ribose polymerase (PARP) inhibitors (PARPi) and bevacizumab have been approved as first-line maintenance therapy. Despite the prolonged progression-free survival, patients did not show a significant long-term survival benefit (Mirza et al., 2020; Shoji et al., 2022). Therefore, increasing treatment trials attempt to extend overall survival.

In recent years, immunotherapy has presented new opportunities. Studies of various cancers have confirmed that immunotherapy significantly improves the outcomes of patients. Emerging Immune checkpoint inhibitors (ICI) rejuvenate CD8^+^ T cells by targeting PD-1, PD-L1, or CTLA4, which can deliver rapid and durable effects for patients with advanced tumors (Mahoney et al., 2015; Van Allen et al., 2015; Sharpe and Pauken 2018). As the treatment of ovarian cancer turns to immunotherapy, new challenges also emerge. The therapeutic of experimental drugs, such as CD274 antibody and CAR-T, are very limited. Ovarian cancer is defined as a “cold” tumor without marked lymphocytic infiltration, which indicates that failure to effectively stimulate the immunity to benefit from the treatment of immunosuppressive checkpoint inhibitors. Immunosuppression networks composed of myeloid-derived suppressor cells (MDSCs), Tregs, TAMs, cancer-associated fibroblasts (CAFs), and adipocytes may negatively affect the immunotherapy (Yang et al., 2020). The tumor immune microenvironment plays a key role in balancing immune escape and immune invasion. Herein, identifying reliable markers based on the relationship between tumor biology and TME characteristics is a crucial development for OC.

Tertiary lymphoid structures (TLS) are ectopic lymphoid organs stimulated by inflammatory signals (Dieu-Nosjean et al., 2014). The character of TLS diverges in different inflammatory atmospheres (Sautes-Fridman et al., 2019; Lutge et al., 2021). When the body is exposed to an infection or tissue damage, TLS invokes powerful immune responses by mobilizing lymphocytes to antigen sediment, while SLO grapples ineffectively (Aloisi and Pujol-Borrell 2006; Moyron-Quiroz et al., 2006). Conversely, TLS would locally generate auto-reactive T and B cells, accelerating the progression of autoimmune disease or incapacitating functions of grafts (Thaunat et al., 2010; Lucchesi and Bombardieri 2013). Increasing evidence substantiated that TLS favorably impacts the prognosis of cancer patients. The superior TLS density positively correlated with overall survival and disease-free survival has been observed in studies of oral squamous cell carcinoma, lung cancer, invasive breast cancer, and colorectal cancer (Gu-Trantien et al., 2013; Di Caro et al., 2014; Germain et al., 2014; Li et al., 2020). As for ovarian cancer, previous studies proposed that TLS coordinates the infiltration of T cells, such as the cytotoxic T cell and CXCL13-producing CD4^+^ T cells, and antibody-producing PCs to advance antitumor responses (Kroeger et al., 2016; Yang et al., 2021; Ukita et al., 2022). However, the impact of TLS on gynecologic malignancies, especially ovarian cancer, demands a better understanding.

Similar to SLO, typical TLSs are organized by T cell zones and B cell zones containing germinal centers (GCs). Multitudinous techniques can be implemented to describe the landscape of TLS. HE staining is the earliest approach to distinguish TLS from morphological mirrors (Barmpoutis et al., 2021). Afterward, various studies clarified the highlights of TLS as a niche for T and B cells responding antagonistically to tumors by performing H&E staining, multi-immunohistochemistry (mIHC), gene expression analysis, and flow cytometry on considerable series of cancer samples (Silina et al., 2018). Nevertheless, there is no unified expert consensus on the presence and quantification of TLS. Subjective factors such as cell markers selected by investigators and working experience in morphological probes increased the experiment bias. Recent research on TLS progressed to the genetic sequencing level. In a study of malignant melanoma, the TLS signature containing nine genes was determined through differential analysis of transcriptome data of B cells and T cells (Cabrita et al., 2020). The gene signature reflected the predicted value of TLS in superior clinical outcomes and better response to ICI therapy. One review constructed TLS hallmark genes by summarizing the TLS features (Dieu-Nosjean et al., 2014). Another study reported a 12-chemokine signature associated with TLS (Lin et al., 2020). Considering the formation of TLS is a combination of lymphocytes and stromal cells under the action of a series of cytokines in the TME, the three gene sets could be generalized as the original collection of TLSs for subsequent analysis.

Therefore, our study was designed to define the appearance and prognostic significance of TLS. Then we established and examined the TLS gene signature of OC strongly correlated to prognosis. Our study discusssed the prognostic impact of the TLS gene signature in OC patients and further examined its predictive value for immunotherapy baed on the immune landscape. The relationship between the gene signature and representative biomarkers was also explored.

## 2 Materials and methods

### 2.1 Data collection and pre-processing

A retrospective study was performed on 60 ovarian cancer patients treated at Qilu Hospital of Shandong University (between 2014 and 2017). The Ethics Committee of Qilu Hospital of Shandong University has approved the collection of experimental specimens. Inclusion criteria were as follows: initial diagnosis of ovarian cancer, unaccepting of neoadjuvant chemotherapy, complete clinical data, and denial of other tumors. We interviewed the patient cohort until 1 June 2022. All patients’ information was anonymized.

RNA-seq and clinical data of ovarian cancer (OC) patients were downloaded from The Cancer Genome Atlas (https://portal.gdc.cancer.gov/). After eliminating the expression profile data with a high degree of variation and incomplete clinical information, finally, 362 patients were selected. Another part of ovarian cancer patients (*n* = 380, GSE140082, GPL14951) from Gene Expression Omnibuswere (https://www.ncbi.nlm.nih.gov/geo) also were recruited for verification.

### 2.2 HE and immunohistochemistry protocol

The HE-stained sections were directly derived from the Pathology Department of Qilu Hospital of Shandong University. After being heated at a stationary temperature of 65° for 1.5 h, paraffin-embedded slides were dewaxed by xylene and immersed in decreasing ethanol concentrations for hydration. We applied heat-induced epitope retrieval for 15 min to expose the antigen. These slices were separately covered in hydrogen peroxide solution and goat serum for 30 min. Primary antibodies (CD20, 1:200, Rabbit mAb, Cell Signaling Technology, United States; CD3, 1:100, Rabbit mAb, Abcam, Cambridge, United Kingdom; CD8, 1:200, ab3516, Abcam, United Kingdom) were used to specifically bind to antigens under 4°C lasting for 12 h. And we applied immunopotentiator and secondary antibody modified by horseradish peroxidase to combine the primary antibody. Samples stained by 3,3′-diaminobenzidine (DAB) and dehydrated under alcohol were continued to be counterstained by hematoxylin. Lastly, these slices were faxed by neutral gum. All images of sections were analyzed by three mature pathological professors. The organized dense lymphocyte aggregates in HE-stained samples were identified as tertiary lymphoid structures. Histology of CD20^+^ B cells and CD3^+^ T cells were evaluated to judge the maturity of TLS. The evaluation criteria are Semi-quantitative of lymphocytes: a. Proportion of lymphocytes in stroma: <5% = 0; 5%–25% = 1; 26%–50% = 2; 51%–75% = 3; 76%–100% = 4; b. The staining intensity: without any staining = 0; faint yellow = 1; claybank or brown = 2; The total scores = a+b. The cohort was divided into high and low groups according to average scores.

### 2.3 The establishment of TLS gene signature of ovarian cancer

There are 25 genes in the three published TLS-associated datasets, but only 21 genes were found in the TCGA-OC expression profile data. These 21 genes were used as a background set (Dieu-Nosjean et al., 2014; Cabrita et al., 2020; Lin et al., 2020). We implemented univariate cox regression analysis to screen the genes signature related to the prognosis of ovarian cancer patients in the TCGA database (*p* < .05). Simple sample Gene Set Enrichment Analysis (ssGSEA) is an algorithm to calculate the enrichment fraction of the pairing of each sample and gene set, whose most common application is to calculate immune infiltration (Barbie et al., 2009; Chen et al., 2022). Mimicking its computational process in immune infiltration, we take the established TLS gene set as the target gene set. The enrichment score of each sample was calculated through the R package “GSEA,” and used as the score of the TLS gene signature. Then the study cohort (the TCGA dataset as train set, the GSE140082 dataset as test set) were divided into high and low expression groups based on median scores.

### 2.4 Analysis of CIBERSORT and ESTIMATE

CIBERSORT and ESTIMATE are the two most common algorithms for assessing immune infiltration. CIBERSORT is a tool for deconvolution of the expression matrix of human immune cell subtypes based on the principle of linear support vector regression (Chen et al., 2018). ESTIMATE is a method developed to evaluate tumor purity by using gene expression characteristics to infer the ratio of stromal cells to immune cells in tumor samples (Yoshihara et al., 2013). We discussed the functional lymphocytes among 22 kinds of immune cells by the R package “CIBERSORT” based on the TLS signature respectively (Newman et al., 2015). And the R package “ESTIMATE” were used to compare the differences in immune scores, stromal scores and ESTIMATE scores between high and low TLS signature groups (Yoshihara et al., 2013).

### 2.5 The analysis of TIDE scores and IPS scores

The developers of TIDE proposed two mechanisms of immune escape: 1) Dysfunction: T cells are inhibited by high levels of CTL; 2) Exclusion: suppression of T cell at low levels of CTL infiltration (Jiang et al., 2018). Higher TIDE score means a greater likelihood of immune escape and thus a poorer response to ICI. Data of OC patients were also downloaded from The Cancer Immunome Atlas (TCIA) (https://tcia.at/home). The IPS score (0–10 score) of each sample was standardized by the gene expression in four antibodies as immunotherapy (Gui et al., 2021). If the IPS score is higher, ICI is more effective.

### 2.6 The prediction of PD-L1 efficacy of the TLS signature

Most solid tumors can be classified into three immunological phenotypes: immune-inflamed, immune-excluded, or immune desert (Hegde et al., 2016). Cohered with the RECIST (1.1) criteria, the patients were assessed as responders with complete remission rate (CR), or partial remission rate (PR), and non-responders with stable disease (SD) or progressive disease (PD). We extracted the expression profile and clinical data from the IMvigor210 dataset, in which patients with locally advanced or metastatic urothelial carcinoma. The nine genes established before were applicated again to excogitate a risk score equation by the same route to compare the differences in risk scores and survival among immune subtypes and response subgroups (Mariathasan et al., 2018).

### 2.7 Mutation and check point genes analysis

The mutation frequency of TCGA-OC patients was calculated by the “maftools” package. These samples were divided into high and low TMB groups based on median of per/MB of mutation. And the onco-plot waterfall plot was analyzed to summarize the details of the single-nucleotide variant (SNV), including the top 10 genes, the base of the mutation site, and types of mutation. Copy number variations (CNV) of samples was also calculated. And we used PD-L1 and CTLA-4 as representatives of immune checkpoint genes to analyze the relationship with TLS gene characteristics.

### 2.8 Statistical analysis

Statistical analyses were conducted using R software (version4.0.3) and SPSS (23.0, IBM). The samples were grouped according to median. The relationship between TLS and clinical characteristics was assessed by chi-square test. Wilcoxon test was employed to analyze differences between two TLS signature groups. Differences in Overall Survival (OS) and Progression-Free Survival (PFS) between different groups were evaluated by Kaplan—Meier survival curve and verified by the log-rank test. The prognostic capability of the emergence of TLS and gene signature were evaluated by singular and multiple Cox regression analysis. A two-sided *p*-value of <.05 was considered statistically significant.

## 3 Results

### 3.1 TLSs are organized structures in ovarian cancer

**FIGURE 1:**
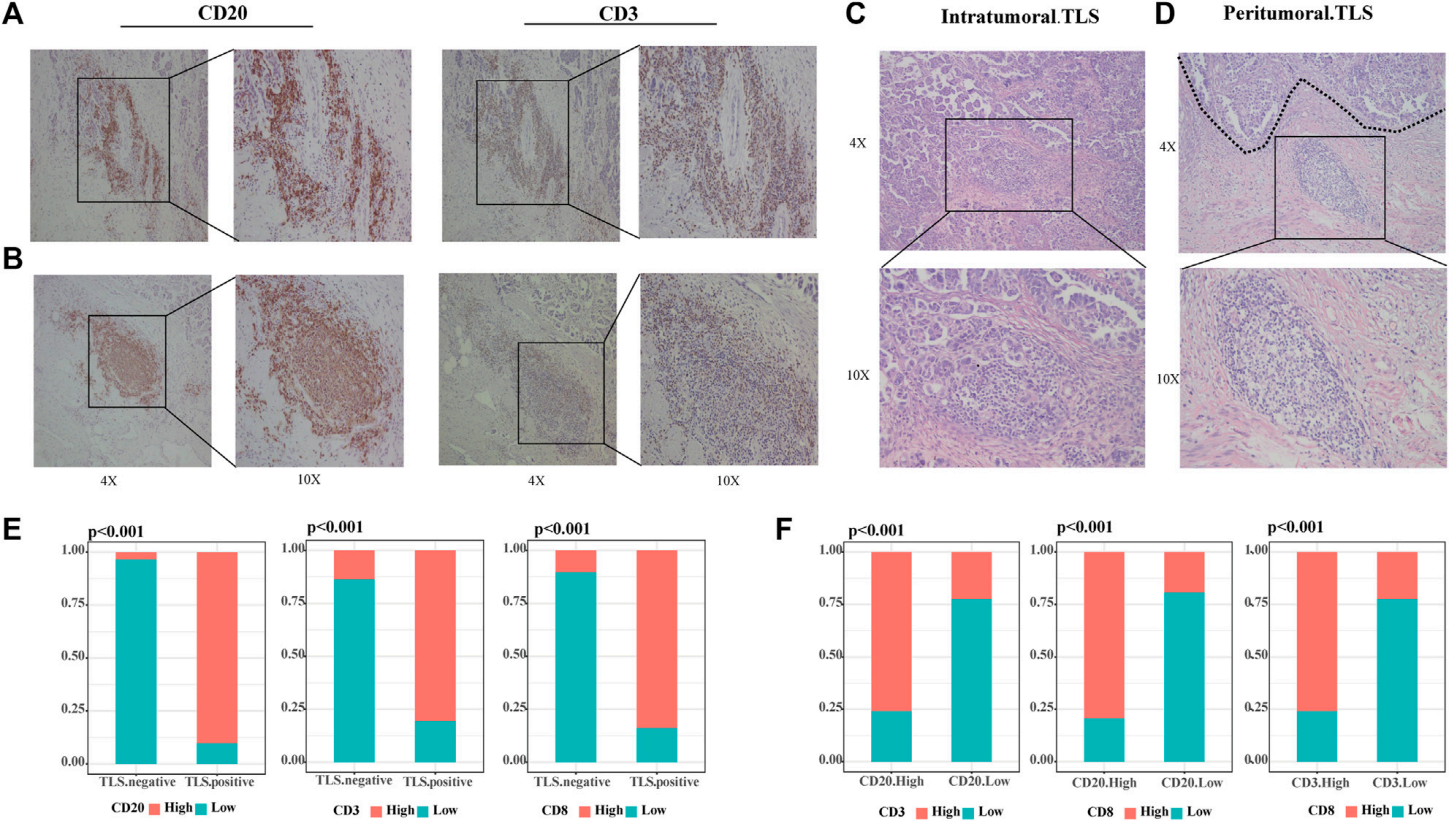
TLSs are dense clusters of lymphocytes in ovarian cancer. **(A)** Representative IHC images of immature TLS, scattered CD20^+^ B cells without any follicular structure and CD3^+^T cells. **(B)** Representative IHC images of mature TLS, CD20^+^ B zone form a follicular structure surrounded by the CD3^+^ T-cell zone. **(C)** Representative HE images of intratumoral TLS, immature TLS. **(D)** Representative HE images of peritumoral TLS, mature TLS. **(E)** Comparison of lymphocyte distribution between TLS-positive and negative groups, *p*-value was determined by the chi-square test. **(F)** Correlation between lymphocyte distribution, *p*-value was determined by the chi-square test. (Scale bar, 200 μm in “4X” pictures, 100 μm in “10X” pictures; *p* < .05).

Our study defined tertiary lymphoid structures (TLSs) as organized clusters of lymphocytes present in HE-stained tumor specimens from OC patients (31/60 36.67%). We categorized TLS into two classes according to the number of CD20^+^ B cells and CD3^+^ T cells, and the formation of lymphoid follicles through IHC images. Scattered lymphocytes were found in TLS-negative specimens. The samples with small concentrations of B and T cells in the stroma are judged of immature TLS (12/31 38.71%) (Figure 1A). When CD20^+^ B cells assemble and evolve into lymphoid follicles, surrounded by CD3^+^ T cells marginal zone, mature TLS (19/31 61.29%) were settled down (Figure 1B). This phenomenon suggested that the maturation of TLS is a dynamic process. TLSs recruit lymphocytes to form dense ectopic tissue at the tumor boundaries. We observed that in TLS-positive samples, most mature TLS were located in the tumor margin (Figure 1C), while immature TLS were nearly shown to be intratumoral TLS (Figure 1D). Compared to TLS-negative cases, the high CD20^+^ B cells, CD3^+^ T cells, and CD8^+^ T cells groups accounted for significantly more in those with TLS (Figure 1E, all *p* < .001). Further, the infiltration of CD20^+^ B cells, CD3^+^ T cells, and CD8^+^ T cells were strongly correlated with each other (Figure 1F, all *p* < .001).

### 3.2 The existence of TLSs represents a superior survival outcome

**FIGURE 2:**
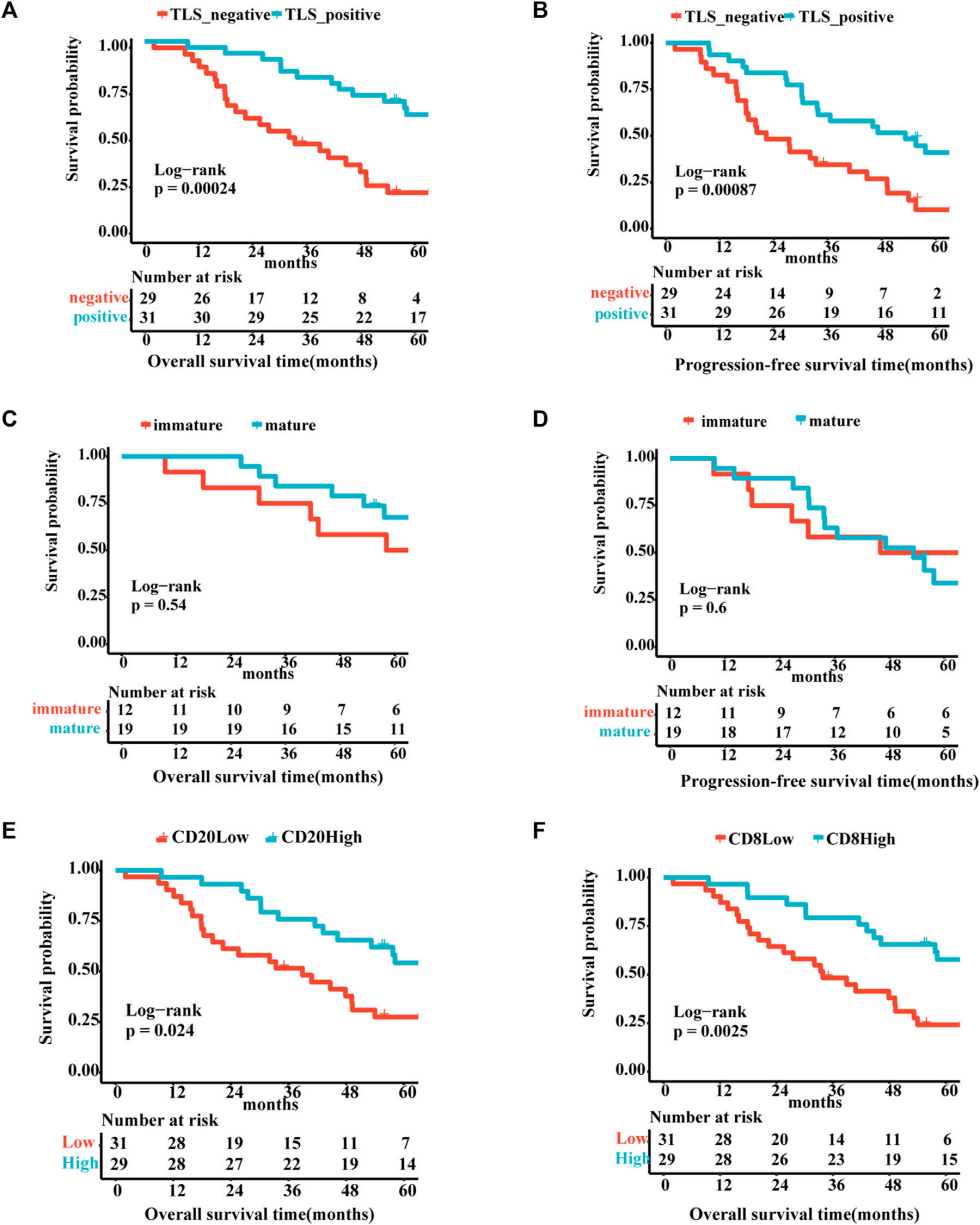
The existence of TLSs represents superior survival outcome Survival analysis of OV patients between the positive and negative TLS subgroups **(A,B)**: **(A)** The 5-year overall survival. **(B)** The 5-year progression-free survival. Survival analysis of OV patients between the immature and mature TLS subgroups: **(C)** The 5-year overall survival. **(D)** The 5-year progression-free survival. Survival analysis of OV patients between the high and low TIL subgroups **(E,F)**: **(E)** The 5-year overall survival of CD20. **(F)** The 5-year overall survival of CD8. (*p* < .05).

The correlation between TLS and clinical factors is shown in Supplementary Table S1. These variables were included in the univariate regression analysis through the log-rank test. Stage I/II and TLS-positive exhibited a favorable trend towards advanced 5-year OS. Furthermore, TLS-positive plays a role of independence in multivariate analysis (Supplementary Table S2). Correspondingly, TLS-positive also is an independent factor for 5-year PFS sequentially by univariate and multivariate analyses (Supplementary Table S3). As portrayed in Figures 2A, B, the TLS-positive group performed the superior 5-year overall survival and progression-free survival (5-year-OS: *p* = .0078; 5-year-PFS: *p* = .041). However, the survival curves showed no significant difference in 5-year OS and 5-year PFS between immature and mature TLS groups (Figures 2C, D, 5-year-OS: *p* = .54; 5-year-PFS: *p* = .6). And the better prognosis of high expression of CD20^+^ B cells and CD8^+^ T cells groups than low expression groups interpreted that TLS achieves an anti-tumor immune response through the synergistic performance of critical immune cells (Figures 2E, F).

### 3.3 The correlation of TLS signature with OS in OV patients

**FIGURE 3:**
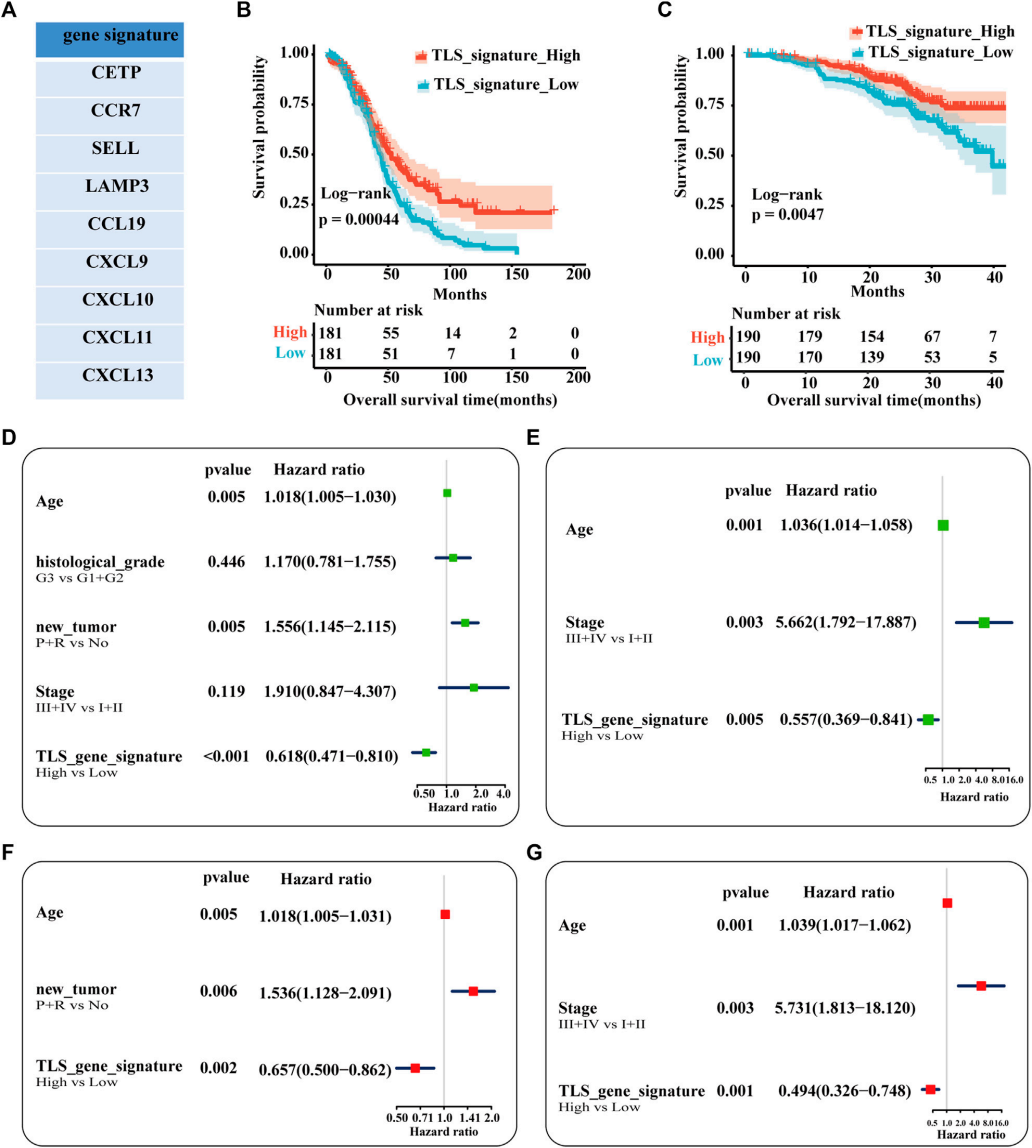
Analysis of the independence of TLS gene signature on prognosis. **(A)** The nine genes associated with better prognosis in TCGA-OV dataset. Survival analysis of OV patients between the high and low TLS signature subgroups **(B,C)**: **(B)** The TCGA cohort as training set. **(B)** The GSE queue as validation set. Univariable analysis **(D)** and multivariable analysis **(F)** of the overall survival in TCGA-OV cohort. Univariable analysis **(E)** and multivariable analysis **(G)** of the overall survival in GSE140082 cohort. (tumor status: No = without-tumor; P = progression; R = recurrence, *p* < .05).

Our study established the gene signature (CETP, CCR7, SELL, LAMP3, CCL19, CXCL9, CXCL10, CXCL11, and CXCL13) of TLS associated with the prognosis of ovarian cancer patients after including 21 genes in univariate regression analysis (Supplementary Figure S1, Figure 3A). Based on the matched scores of the genes set, OC patients were separated into TLS signature high (above 50%) and low queues (below 50%). As exhibited in Figure 3B, the high expression group presented a superior trend towards improved OS (*p* = .00044). Univariate and multivariate regression analyses were evaluated by comparing the effect of the gene feature with other parameters on the overall survival of patients. Age, the recurrence and progression of the tumor, and TLS gene signature were screened as a statistically prognostic factor in univariate analysis (Figure 3D). Furthermore, the TLS signature was associated with a significantly better OS in multivariate analyses (Figure 3F). Considering the lack of significance of tumor stage in the prognosis of OC patients in the TCGA-OC database, we used the GSE140082 database as the validation cohort. Likewise, the TLS gene signature was also an independent predictor of OS (Figures 3C, E, G). Besides, the prognostic significance of this TLS gene signature in PFS is illustrated in Supplementary Figure S2.

### 3.4 The relationship between TLS signature and immune cells infiltration

**FIGURE 4:**
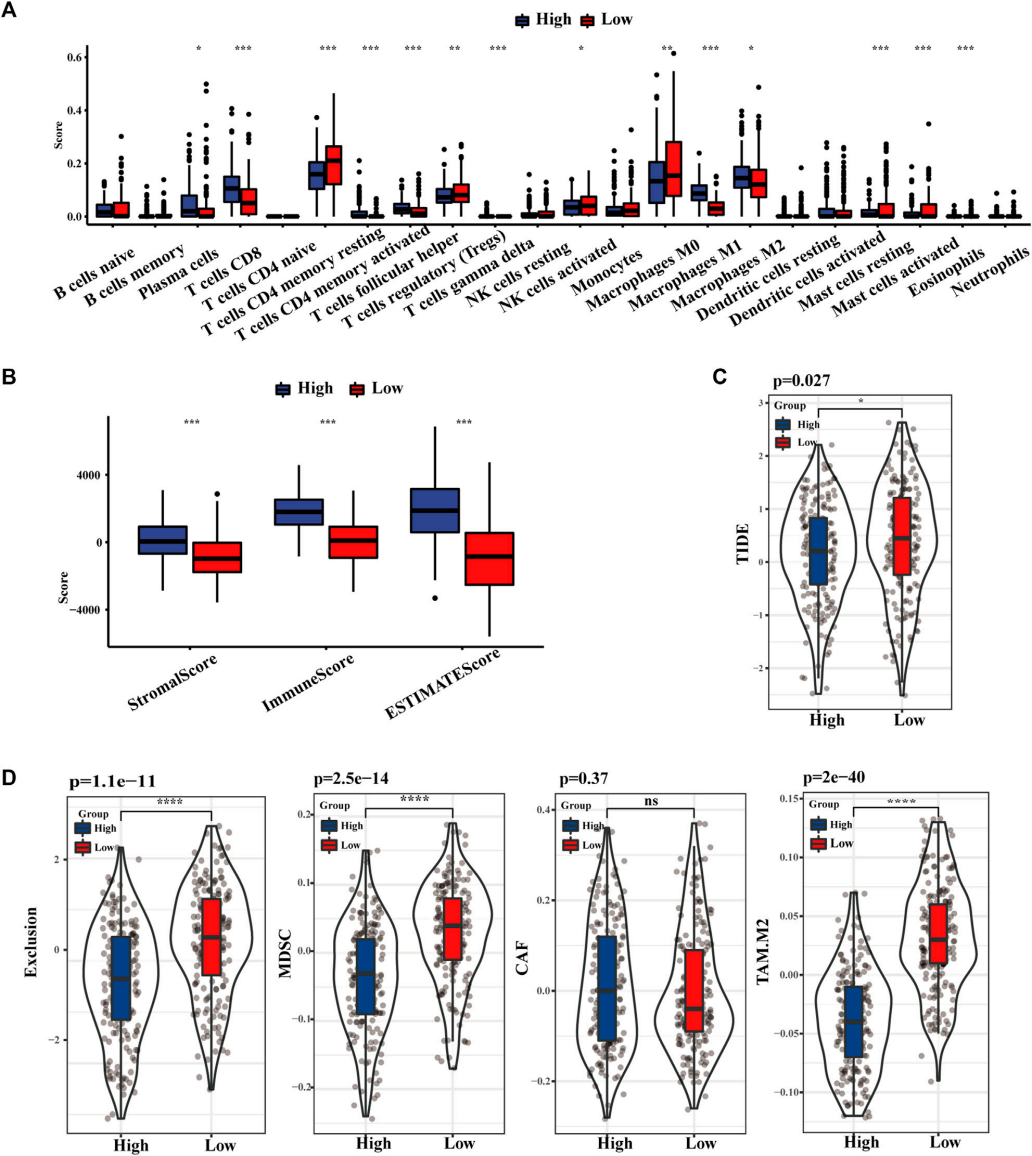
The landscape of Immune infiltration based on TLS signature. **(A)** The distribution of 28 immune cells between high and low TLS signature groups. **(B)** Box plots of TLS signature score in stromal, immune, and estimate score. **(C)** Violin plot of the differences in calculated TIDE scores between high and low TLS signature groups. **(D)** Differences in immune escape mechanisms and immunosuppressive cells between the two groups of TIDE. (NS: no significance, ∗*p* < .05, ∗∗*p* < .01, and ∗∗∗*p* < .001).

As displayed in Figure 4A, the distribution of 22 kinds of immune cells differs with the TLS signature subgroups. The degree of CD8^+^ T cells, activated and resting memory CD4^+^ T cells, regulatory T cells (Tregs), and M1 phenotype macrophages in the high TLS expression group was statistically higher than in the low group. Similarly, the TLS signature high group obtained distinctly higher immune score, stromal score, and estimate score, which indicated that gene expression levels associated with TLS prominently affect the infiltration situation of immune cells and stromal cells (Figure 4B). Correspondingly, the TIDE score in the low-risk group was notably higher than in the lowgroups, indicating the existence of tumor immune evasion in the low-risk group (Figure 4C). We further discovered that the immunosuppressive cell expression models, namely T cell exclusion, dominated the immune escape of the low TLS signature group. Meanwhile, the degree of IFNG, CD274, and CD8 in the high TLS signature group was significantly higher than in the low TLS signature group (Supplementary Figure S3). Conclusively, our study indicated the high TLS signature group as the cohort most potentially benefiting from immunotherapy (Figure 4D).

### 3.5 The predictive value of TLS features in immunotherapy response

**FIGURE 5:**
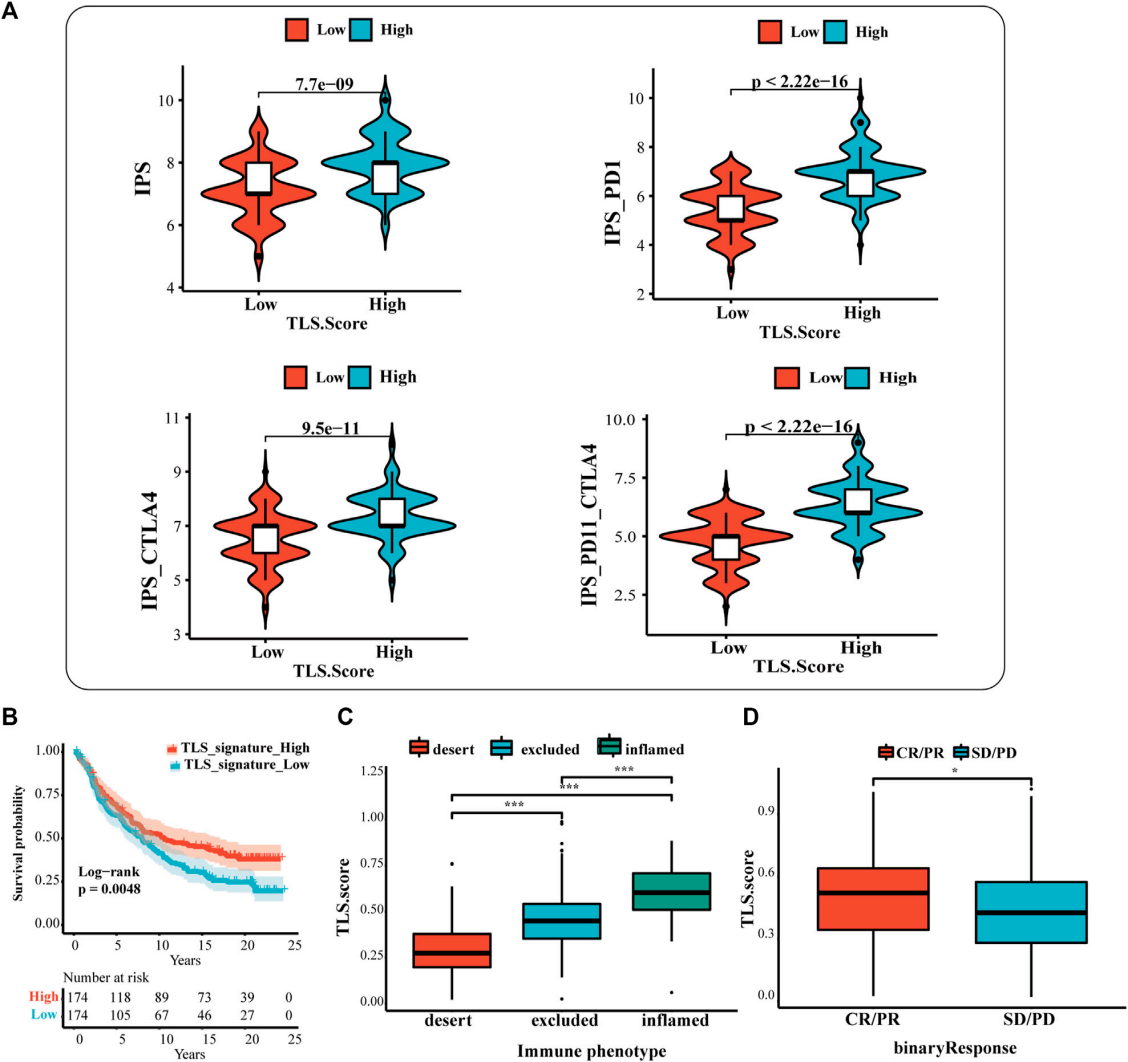
The prognostic value of TLS features in immunotherapy response. **(A)** The association between IPS analysis and TLS signature score. The analysis of TLS signature in the IMvigor210 cohort **(B,D)**: **(B)** The survival analysis between the high and low TLS signature subgroups; **(C)** Box plots of TLS signature score in the desert, excluded and inflamed immune phenotypes; **(D)** Distribution of ISS in groups with different anti-PD-L1 clinical response statuses. (NS: no significance, ∗*p* < .05, ∗∗*p* < .01, and ∗∗∗*p* < .001).

The results showed that the IPS, IPS-CTLA4, IPS-PD1, and IPS-PD1-CTLA4 scores were higher in the high TLS signature group (Figure 5A). Additionally, the difference reinforces the better response of the high-expression group to immunotherapy. Based on the above results, we applied the TLS signature to the urothelial carcinoma patients who received the treatment of PD-L1 inhibitors (at-eculizumab) to inspect the estimation of TLS signature in ICI. The results displayed that the high TLS signature exhibited the prognostic value (Figure 5B). Meanwhile, the prediction of the TLS signature in immunological phenotype was investigated. In this cohort, the TLS signature score of immune-inflamed phenotype was more distinguished than that of the immune-desert or the immune-excluded phenotype (Figure 5C). Then, we further discussed the differences in PDL1 immune checkpoint inhibitor benefits between high- and low TLS signature groups. It turned out that patients in the high group performed a higher complete response (CR)and partial response (PR) rate (Figure 5D).

### 3.6 The relationship between TLS gene signature and existing markers of immunotherapy

**FIGURE 6:**
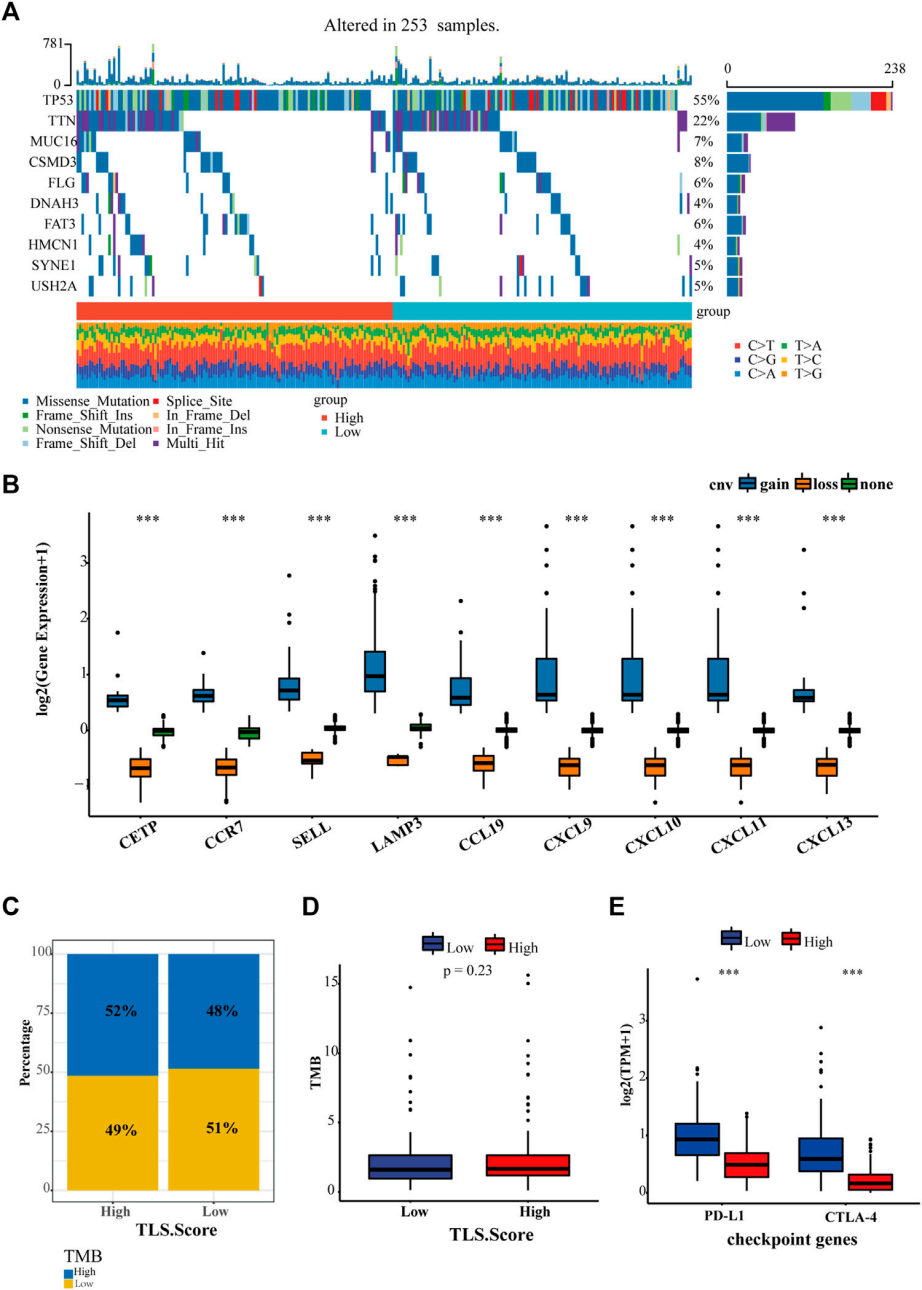
The relationship between TLS gene signature and existing markers of immunotherapy. **(A)** The landscape of genetic variation of the TLS gene signature in the OV cohort. **(B)** The CNV variation frequency of the TLS gene signature in the OV cohort. The proportion **(C)** and frequency **(D)** of tumor mutation load between high and low TLS gene signature groups. **(E)** The distinction of PD-L1 and CTLA-4 between high and low TLS gene signature groups. (∗*p* < .05, ∗∗*p* < .01, and ∗∗∗*p* < .001).

Except for tumor-infiltrating lymphocytes, tumor mutational burden (TMB) and immune checkpoints, such as PD-1, PD-L1, and CTLA4, have been developed to predict immunotherapy. Hence our study proceeded to estimate the diversity between the landscape of tumor mutation and typical checkpoint genes between high and low TLS signature groups. In total, 235 samples were genetically altered. The top 10 genes with mutation frequency between TLS high group and the low group were generalized in Figure 6A. The most common variant classification is a missense mutation. SNP ranks first in variant type, and C>A is the most frequent SNV class (Figure 6A, Supplementary Figure S4). In addition, we found that the gene signature was characterized by a precise copy number amplification (Figure 6B). The high TLS signature group occupied an increased proportion of high-TMB than the low group, despite the statistically significant difference in TMB between the two groups being not shown (Figures 6C, D). Moreover, the expression of two representative checkpoint genes, PD-L1 and CTLA4, significantly increased in the high TLS signature group (Figure 6E).

## 4 Discussion

Considering that the effect of immunotherapy on ovarian cancer is restricted, exploring biomarkers or targets to filtrate ideal beneficiaries is a future development. The three objectives of this study are as follows. First and foremost was to assess the presence of TLS and its prognostic impact on ovarian cancer. Identification of an independent signature of TLS associated with prognosis is the second goal. The third aspect is to explore the immune landscape and predictiveness of immunotherapy of this gene signature.

Recently the remarkable manifestation of tertiary lymphatic structures in the tumor microenvironment has begun to be noticed. Various studies on neoplasms have confirmed the prognostic value of TLS (Cabrita et al., 2020; Vella et al., 2021). Consistent with their results, our study revealed that TLS is strongly associated with prolonged OS and PFS. Beyond that, the higher density of CD20^+^ B cells, and CD8^+^ T cells play an excellent role in advanced 5 years overall survival. These data suggest that TLSs exert efficient antitumor response by recruiting activated B cells and T cells in TME. Tumor-infiltrating lymphocytes accumulate orderly in ectopic organs to form TLS, but the specific antitumor immune reactivity remains obscure. As the most abundant cells in TLS, B cells progressively enrich into GC as TLS develops. Moreover, B cells within GC tend to mature into the IgG and IgA-producing plasma cells (PCs), which can migrate along the fibroblast track to the nest of tumor (Meylan et al., 2022). Driven by memory B cells and a range of cytokines (including TNF, IL-2, IL-6, and IFNγ), T cells home to the tumor bed and release perforin and granzyme or Fas/FasL pathways to destroy tumor cells. However, the single presence of TIL was suboptimal. Researches confirmed that tumor-infiltrating CD8+T cells showed their cytotoxic capacity function only when B cells were enhanced within the tumor (Kroeger et al., 2016). The simultaneous occurrence of intratumoral CD8^+^ T cells and CD20^+^ B cells was also found to be independently associated with improved survival (Wouters and Nelson 2018; Cabrita et al., 2020). Noticeably, the maturation stage of TLS could significantly affect the prognosis in theory, but this study found no significant imparity between immature and mature TLS groups. Finite-sized and single-center samples of this study may contribute to the dispute. However, these results showed that the tumor immunology advantage of TLS is that it increases the opportunity of binding tumor antigen by the recruitment of TIL, while serving as a collection point, strengthening the interaction between TIL and cytokines. For immune-deficient ovarian cancer, TLS brings positive forces for increasing sensitivity to ICI. TLS-related drugs or vaccines can improve the effect of ICI under development at OC.

To date, there is no unified expert consensus on the identification and quantification of TLSs. Consistent with previous studies, this study also conducted H&E staining and immunohistochemistry (IHC) with selected markers to detect TLS, while these measures are inconvenient and subjective. In the context that TLS is a combination of lymphocytes and cytokines, this study innovatively selected and constructed the TLS signature related to prognosis in ovarian cancer from background genes. Satisfactorily, ovarian cancer patients in public datasets with high TLS signature performed a preferable survival outcome. The established genetic traits include CETP, CCR7, SELL, LAMP3, CCL19, CXCL9, CXCL10, CXCL11, and CXCL13. CXCL13 acts as a B lymphocyte chemoattractant in TLS, mediating the recruitment of B cells and promoting T/B separation and the formation of a germinal center (GC) within TLS (Rodriguez et al., 2021). The consequence of CXCL13 in ovarian cancer is gradually understood. A large study of high-grade serous ovarian cancer found CXCL13 enhanced immune efficacy in combination with PDL1 by promoting the expansion and activation of CXCR5 + CD8 + T cells. We also observed that the CXCL13-producing CD4 + T cells could facilitate the formation of TLS and strengthen the cooperative antitumor activity of cellular and humoral immunity in ovarian cancer (Ukita et al., 2022). CXCL9, CXCL10. CXCL11/CXCR3 axis contains the four genes of this gene signature. The excellent performance of the axis in TME reflects in the migration, differentiation, and activation of immune cells. In *in vitro* experiments of mice, as the downregulation or inhibition of the expression of the CXCR3 axis, the migration of Th1, NK, and CTL cells were all significantly decreased, and the survival outcome also worsened (Tokunaga et al., 2018; House et al., 2020). In high density of LAMP3-DC-mature-type TLS, CXCL9, 10 and 11 are involved in the migration of Th1 cells by binding to the CXCR3 receptor on tumor-infiltrating T cells (Sautes-Fridman et al., 2019). A sequencing analysis for 1,310 breast cancer patients demonstrated the relevance of CXCL10 and HRD, identifying CXCL10 as biomarker for anti-PD-1/PD-L1 therapy (Shi et al., 2021). Paradoxically, this axis induces the onset of immunosuppression by attracting Treg migration to the focal site. In the context of the contribution of HRD in ovarian cancer, CXCL10 provides a neoteric perspective on markers of immunotherapy. Improved survival, a preferable anti-PD-L1 therapy caused by the overexpression of CXCL9 in ovarian cancer, determined it as a stable predictive target (Seitz et al., 2022). The role of CETP, CCR7, and SELL in ovarian cancer is less studied. The chemokine ligand CCR7 and the adhesion molecule CD62L (expressed by the SELL, L-selectin) act as lymph node homing receptors that regulate T cells’ activation and migration patterns. The loss of KLF2 resulted from the activation of PI3K and mTOR and subsequently prevented the target genes, such as CD62L, and the reduction of CCR7 (Sinclair et al., 2008). L-selectin shedding and transcriptional shutdown allow terminal differentiation of naive T cells to effector memory T cells (TEM), while TEM flow from secondary lymphoid organs to peripheral tissues for repositioning (Ivetic et al., 2019). And the downregulation of KLF 2 and its target gene S1PR1 antagonistically prompted the upregulation of the type-C lectin CD69. Meanwhile, the locally produced TGF-βis all involved in the expression of CD103, leading to the retention of T cells in the tissue, namely the generation of tissue-resident T cells (TRM). Workel et al. (2019) found that TGF-βprompted CD8CD103^+^ T cells to secrete CXCL13, which recruits B cells in TME and is essential for the formation of TLS. Therefore, we hypothesize that there is a strong association between the expression of SELL and CCR7, TRM, and TLS, and exploring their interactions may provide new ideas for tumor treatment. For example, a new immunotherapy concern is restoring control of selectins to modulate tumor immune expansion. In a mouse model of adoptive T cell cancer immunotherapy, overexpression of L-selectin improved the control of tumor cells by promoting infiltration and proliferation of T cells (Watson et al., 2019). Retrospecting the existing achievement, this gene signature is feasible for predicting prognosis and immunotherapy efficacy. However, the specific mechanisms and how to adequately activate its potency in immunotherapy still have a long way to go.

As noted above, tumor-infiltrating lymphocytes are extensively involved in the antitumor response of TME and are considered a positive indicator of the prognosis and efficacy of immune checkpoint inhibitors (Germain et al., 2014). Our data discovered that the percentage and distribution of immune cells is positively correlated with the expression level of the TLS gene signature. And interestingly, this study also found that immunosuppressive subsets also occupied a high proportion in the TLS high expression group. This result revealed that immunosuppressive subsets are also essential parts of TLSs. TLSs undertake the regulator in balancing immune infiltration and immune tolerance of TME. One must be vigilant that TLS may also produce auto-reactive lymphocytes with self-mediated immune toxicity, which cripple immune checkpoint blockade.

PD-L1 has been reported to be negatively correlated with prognosis in malignancy (Nakanishi et al., 2007). Atezolizumab, as the PD-L1 inhibitor, is currently the only FDA approved for BCa treatment. Combined with TIDE, IPS analysis, we found that TLS signature is correlated with a better response to ICB. The high TLS signature group are determined as a potentially beneficial population for TLS immunotherapy after verification in BCa data. Considering the ICB only provides remission in a small percentage of patients (Gong et al., 2018), the TLS signature developed by our study would help clinicians stratify patients to select those with potential benefit from ICB. Due to the lack of public data on ovarian cancer immunotherapy, the accuracy of this feature prediction is subject to further debate. Now molecular profiling that guides ovarian cancer immunotherapy includes tumor mutational burden (TMB), homologous repair deficient and proficient (HRD, HRP) phenotypes, neoantigen intratumoral heterogeneity (ITH), and tumor-infiltrating lymphocytes (TILs) (Yang et al., 2020; Morand et al., 2021). Considering the ovarian cancer is a “cold” tumor without high TMB, combining TMB and other potential markers is redefined as necessary to improve the predictive ability. Interestingly, this study discovered that the mutation burden, PDL1 and CTLA4 in high TLS gene signature group are higher than in the low group. So, these could be applied to the combined prediction of OC. As for HRD, it is mainly used to predict the response to poly-(ADP ribose) polymerase (PARP) inhibitors and platinum chemotherapy in OC. Several groups have generalized the HRD gene set, including MMR, BRCA1/2, POLD1 or POLE, MUTYH, and ERCC1 (Zhang et al., 2020). Research on the relationship between mutational details of these genes and TLS may lead to new directions for OC immunotherapy.

## 5 Conclusion

This study proved the existence of TLS s and its application as a positive prognostic marker in ovarian cancer. And we constructed an ovarian cancer-associated TLS gene signature, which has a high predictive value in the prognosis and response to immunotherapy.

## Data Availability

The datasets presented in this study can be found in online repositories. The names of the repository/repositories and accession number(s) can be found in the article/Supplementary Material.
