# Supplementary material for: The gene signature of tertiary lymphoid structures within ovarian cancer predicts the prognosis and immunotherapy benefit
Source: Front Genet. 2023 Jan 10;13:1090640. doi: 10.3389/fgene.2022.1090640 (PMC9871364; doi:10.3389/fgene.2022.1090640)
Supplement: Supplementary file 1 [file DataSheet1.docx]

Supplementary Material

# Supplementary Tables

**Table S1** The correlation of TLS and clinical factors.

| Variables |  | TLS-positive  (N=31) | TLS-negative  (N=29) | P |
| --- | --- | --- | --- | --- |
| Age | <=55 | 15 | 13 | 0.782 |
|  | >55 | 16 | 16 |  |
| Histology grade | low | 8 | 6 | 0.640 |
|  | high | 23 | 23 |  |
| Tumor size | <=5cm | 6 | 10 | 0.185 |
|  | >5cm | 25 | 19 |  |
| Stage | I/II | 12 | 8 | 0.361 |
|  | III/IV | 19 | 21 |  |
| Survival outcome | death | 14 | 23 | <0.001* |
|  | live | 17 | 6 |  |
| CD20 | low | 3 | 28 | <0.001* |
|  | high | 28 | 1 |  |
| CD8 | low | 5 | 26 | <0.001* |
|  | high | 26 | 3 |  |

TLS, tertiary lymphoid structure; OS, overall survival; * P<0.05.

| Variables | | Univariate | | Multivariate | |
| --- | --- | --- | --- | --- | --- |
|  |  | HR(95% CI) | P | HR(95% CI) | P |
| Age | <=55 | Referent |  |  |  |
|  | >55 | 1.06(0.55,2.01) | 0.869 |  |  |
| Histology grade | Low | Referent |  |  |  |
|  | High | 5.10(1.971,13.2) | 0.729 |  |  |
| Tumor size | <=5cm | Referent |  |  |  |
|  | >5cm | 1.12(0.53,2.38) | 0.766 |  |  |
| Stage | I/II | Referent |  |  |  |
|  | III/IV | 5.10(1.97,13.20) | <0.001* | 5.06(1.94,13.18) | 0.001* |
| TLS | positive | Referent |  |  |  |
|  | negative | 0.82(0.05,13.33) | <0.001* | 3.338(1.67,6.69) | 0.001* |

**Table S2** Univariate and multivariate 5-year overall survival analysis.

TLS: tertiary lymphoid structure; OS, overall survival; * P<0.05.

**Table S3** Univariate and multivariate 5-year disease free survival analysis

| Variables | | Univariate | | Multivariate | |
| --- | --- | --- | --- | --- | --- |
|  |  | HR(95% CI) | P | HR(95% CI) | P |
| Age | <=55 | Referent |  |  |  |
|  | >55 | 1.19(0.66,2.13) | 0.568 |  |  |
| Histology  grade | Low | Referent |  |  |  |
|  | High | 1.50(0.74,3.03) | 0.258 |  |  |
| Tumor size | <=5cm | Referent |  |  |  |
|  | >5cm | 1.36(0.68,2.69) | 0.383 |  |  |
| Stage | I/II | Referent |  |  |  |
|  | III/IV | 1.95(1.03,3.77) | 0.048* | 1.95(1,3.79) | 0.050 |
| TLS | positive | Referent |  |  |  |
|  | negative | 2.75(1.48,5.10) | 0.001* | 2.77(1.48,5.18) | 0.001* |

TLS, tertiary lymphoid structure; PFS, Progression-free survival; * P<0.05.

# Supplementary Figure
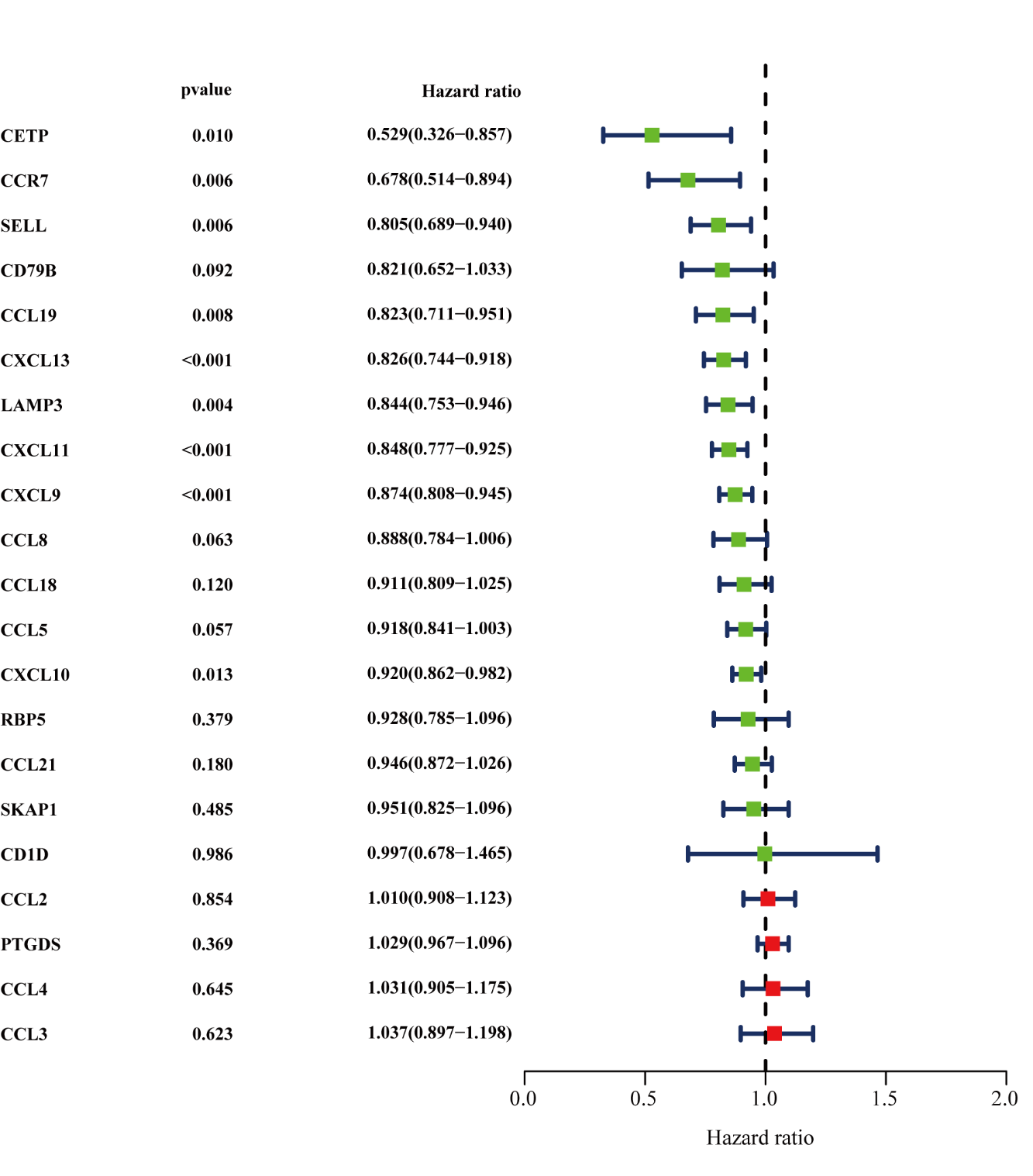


**Figure S1.** Univariate analysis of 21 TLS background genes. p<0.05.


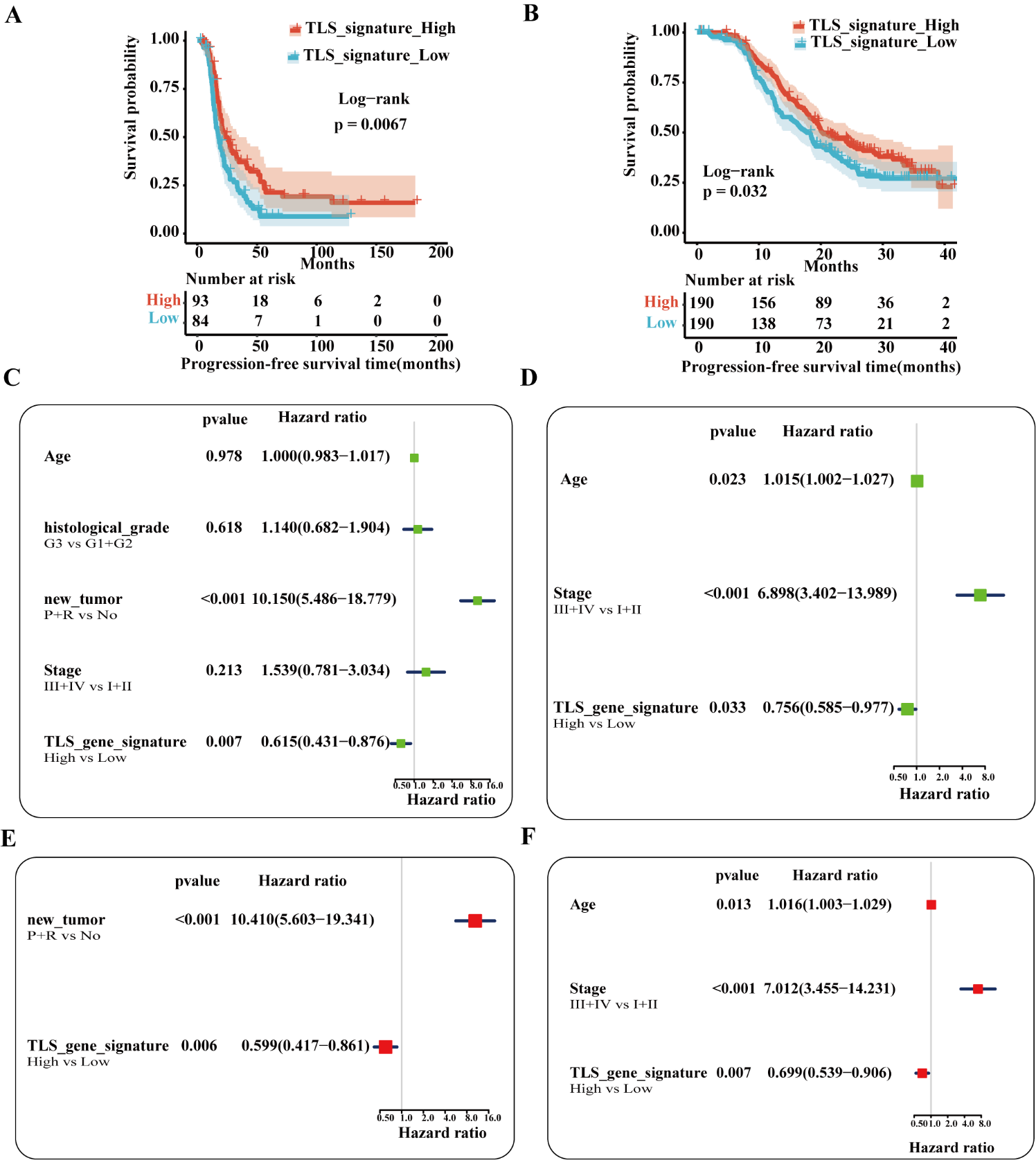


**Figure S2 Analysis of the independence of TLS gene signature on prognosis** Survival analysis of OV patients between the high and low TLS signature subgroups (**A-B**): (**A**) The TCGA cohort as the training set. (**B**) The GSE queue the validation set. Univariable analysis (**C**) and multivariable analysis (**E**) of the progression-free survival in the TCGA-OV cohort. Univariable analysis (**D**) and multivariable analysis (**F**) of the progression-free survival in the GSE140082 cohort. (tumor status: No = without-tumor; P= progression; R= recurrence, p<0.05)


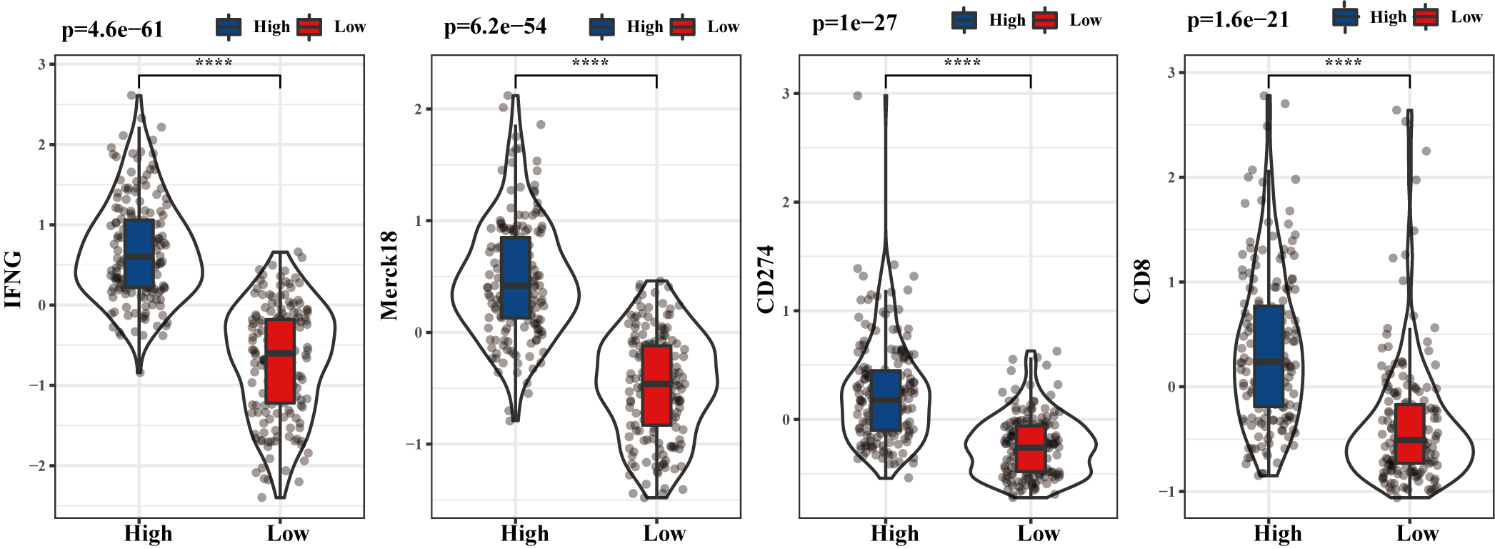


Figure S3 Lymphocytes account for a higher proportion in the high TLS signature group. ∗p < 0.05, ∗∗p < 0.01, and ∗∗∗p < 0.001.

..

.


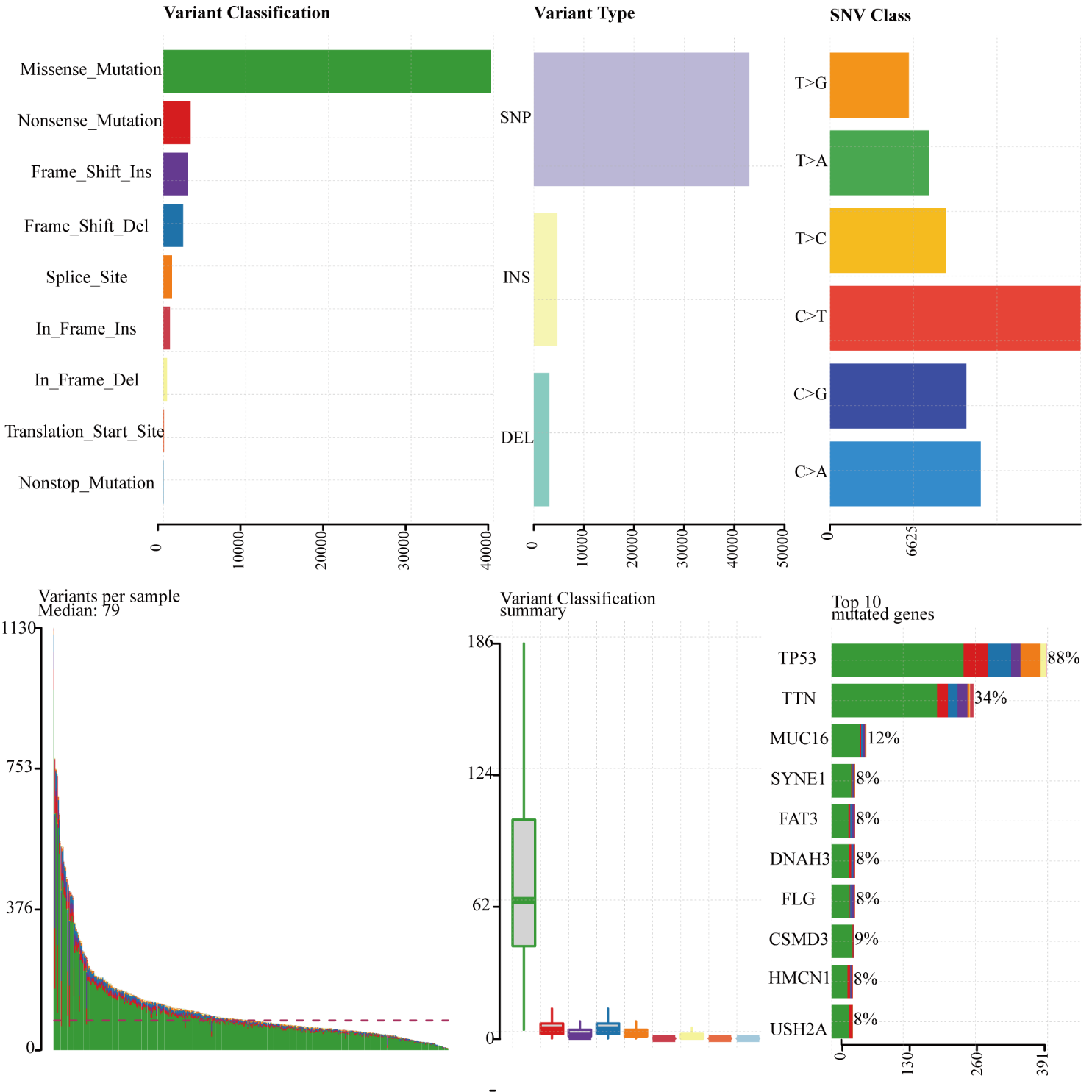


Figure S4 The results of SNV based on the TLS gene signature.
